# Recommendations for Developing a Telemedicine Strategy for Botswana: A Meta-Synthesis

**DOI:** 10.3390/ijerph20186718

**Published:** 2023-09-06

**Authors:** Benson Ncube, Maurice Mars, Richard E. Scott

**Affiliations:** 1 Department of TeleHealth, School of Nursing & Public Health, College of Health Sciences, University of KwaZulu-Natal, Durban 4041, South Africa; mars@ukzn.ac.za (M.M.); ntc.ehealthconsulting@gmail.com (R.E.S.); 2 Department of Community Health Sciences, Cumming School of Medicine, University of Calgary, Calgary, AB T2N 4Z6, Canada

**Keywords:** eHealth, digital health, strategy, telemedicine strategy, eHealth Strategy Development Framework (eHSDF), Botswana

## Abstract

Botswana is developing its eHealth capacity using a National eHealth Strategy. However, that strategy overlooks telemedicine, a potential solution for many healthcare challenges. For telemedicine to benefit Botswana, a telemedicine-specific strategy is required. While establishing a national strategy is a sovereign responsibility, guidance and recommendations can be provided. Using published resources specific to Botswana, key health-related issues were identified. These issues were matched with suitable telemedicine activities and delivery approaches. Recommendations were then derived from these for use in an effective telemedicine-specific strategy for Botswana. From 28 health-related issues, 6 were prioritised. Three telemedicine activities were identified (clinical services, education, and behaviour change), and one delivery approach was chosen (store-and-forward) because they align well with current needs, infrastructure, and mobile phone user capabilities. Since telemedicine has been proven to be effective, efficient, and cost-effective when implemented correctly, this knowledge and experience, plus a recognised strategy development framework, has led to evidence-based recommendations. Specific telemedicine options were identified as examples. As confidence grows, telemedicine options can be expanded to address additional clinical needs and explore alternative activities and delivery options. The recommendations can help the government develop a telemedicine-specific strategy that aligns with the National eHealth Strategy while actively promoting and supporting the adoption and system integration of straightforward telemedicine interventions. This foundation will facilitate the future expansion of telemedicine initiatives for the benefit of all Batswana.

## 1. Introduction

The World Health Organization has, since 2005, clearly indicated that eHealth (the use of information and communication technologies for health), now termed digital health, is an essential component of achieving all 17 Sustainable Development Goals, Universal Health Coverage [1], and for health system strengthening overall [2]. The breadth of the potential impact of eHealth is clear from its definition: “The cost-effective and secure use of information and communications technologies in support of health and health-related fields, including *health care services*, *health surveillance*, *health literature*, *and health education, knowledge, and research*” (italics added for emphasis) [1]. Equally, its importance is emphasised through the WHO’s triple billion targets: “Ensuring that 1 billion more people benefit from universal health coverage, that 1 billion more people are better protected from health emergencies, and that 1 billion more people enjoy better health and well-being” [1].

Telemedicine (a sub-component of eHealth) has been defined as “The delivery of health care services, where distance is a critical factor, by all health care professionals using information and communications technologies for the *exchange of valid information* for *diagnosis*, *treatment and prevention of disease and injuries*, *research and evaluation*, *and the continuing education of health care workers*, with the *aim of advancing the health of individuals and communities*” (italics added for emphasis) [1]. Telemedicine can and has been used for a wide variety of purposes: providing access to rural and remote locations, targeted health communication, decision support, client-to-provider communication, provider-to-provider consultations, tracking health status, delivery of healthcare services, and provision of training and educational content [2]. However, despite this broad use eHealth/Digital health and components remain underused; indeed, when speaking of digital technologies for health, the WHO comments: “Even so, its [digital technologies] application to improve the health of populations remains largely untapped, and there is immense scope for the use of digital health solutions” [3].

As a consequence, developing countries are again responding to the WHO call for national eHealth, now digital health, strategies: “To realize their potential, digital health initiatives must be part of the wider health needs and the digital health ecosystem and *guided by a robust strategy* that integrates leadership, financial, organizational, human, and technological resources and is used as the basis for a costed action plan which enables coordination among multiple stakeholders” (italics added for emphasis) [1].

The literature provides four detailed approaches to eHealth Strategy development [4,5,6,7,8], with guidance regarding national digital health strategies largely restricted to proposed actions from the WHO [1]. These approaches are: the Commonwealth workbook [4], the WHO/ITU toolkit [5], the Tanzanian Case Study [6,7], and the eHealth Strategy Development Framework (eHSDF) [8]. Given the profile and influence of the WHO, most low- and middle-income countries adopt the WHO National eHealth Strategy Toolkit, which states that it provides guidance on “establishing a national eHealth vision, developing a national eHealth action plan, and monitoring and evaluation” [5]. This includes Botswana, where the National eHealth Strategy was released in March 2020 [9].

A review of this national strategy identified an omission that would prevent this sparsely populated country from leveraging the benefits of telemedicine to address the country’s health-related issues (HRI) [10]. Botswana’s eHealth strategy is silent on the structured, sustained, and scaled use of telemedicine (which can include mHealth applications), which should play a major role in developing countries [11], particularly following the COVID-19 pandemic [12]. To address this, the development of a telemedicine-specific strategy that could align with and complement the existing National eHealth Strategy for Botswana has been recommended [10].

Recent research emulated the application of the eHealth Strategy Development Framework [13,14]. It showed that within Botswana, specific knowledge and insight of eHealth and telemedicine amongst healthcare workers and patients were lacking, but there was general acceptance, willingness to use, and belief that telemedicine was helpful [14]. Key Informants also identified priority health issues and proposed solutions to each identified HRI [13]. Some suggested solutions involved eHealth broadly, or telemedicine applications specifically, with three dominant eHealth categories being endorsed: telehealth (including telemedicine), health informatics, and eLearning.

It is against this backdrop that the provision of recommendations for a telemedicine-specific strategy to complement Botswana’s National eHealth Strategy is proffered. Such a supplementary phenomenon is not new, with South Africa [15] and Ethiopia [16] having developed such post hoc strategies, although these are not yet reported to be implemented. However, given that only a national government can develop a binding strategy for its country, this paper presents only ‘recommended’ telemedicine interventions (and the rationale for their selection) which should be considered by the government of Botswana as content for their own telemedicine-specific strategy.

The existing evidence of telemedicine activities within Botswana suggests, with few exceptions, a history of uncoordinated and fragmented deployment [10]. A new strategic approach that will assist in guiding telemedicine initiatives in Botswana is required. Without a deliberate strategy, the ad hoc application of telemedicine solutions and often resultant occurrence of “pilotitis” will remain, and patients and the country will be deprived of improved quality of care and services that could be experienced through thoughtful and structured utilisation of telemedicine.

This study, in accordance with Step 8 of the eHSDF, collates the evidence-base and perceived needs and solutions for telemedicine in Botswana and formulates defensible recommendations for a telemedicine-specific strategy to align with and enhance Botswana’s existing National eHealth Strategy.

### eHSDF Summary

The eHSDF was created for the development of ‘eHealth’ strategy [8]. However, given that telemedicine is a sub-component of eHealth ([17]; Figure 13.1), the same approach is appropriate for telemedicine-specific strategy development and requires only a re-focussing on telemedicine solutions rather than eHealth solutions more broadly (e.g., health informatics, ecommerce, telehealth, telemedicine). The eHSDF is grounded on seven guiding principles and consists of a detailed step-by-step process to be followed to develop an “evidence-based and defensible eHealth Strategy for any specific jurisdiction” [8]. The first seven steps allow for the development of the content of an eHealth strategy, with Step 8 being the synthesis of the evidence gathered in the earlier steps to yield the eHealth strategy itself. Thereafter, Step 9 addresses the eHealth policy required to facilitate the implementation of the strategy.

Steps 1–3 constitute the major evidence-gathering steps and embrace the principle of identifying health needs within the jurisdiction of interest. As early as 1998, the importance of identifying a clear and specific health need for a telemedicine service was noted as a prerequisite, and it has been re-iterated by many authors using various terms over the intervening years [18,19]. This can only be achieved if the prevailing context is thoroughly understood, as achieved through Steps 1 and 2, and refined by Step 3 [8]. Failure to understand health needs can result in telemedicine services not targeting a population’s current primary health concerns, as has been shown in Latin America [20], jeopardising sustainability. Once identified, not all needs can be addressed (given time, technology, and resource constraints), and therefore focus must be given to the most pressing issues, requiring prioritisation of health needs (Step 4).

The aim of the study was to answer the question: “What telemedicine options can be recommended for a telemedicine-specific strategy to address identified priority health-related issues in Botswana?” In keeping with earlier research, the eHSDF was adopted as the guiding theory and approach for strategy development [8].

## 2. Method

### 2.1. Overview

This study adopts the principles of meta-synthesis (review and integration of findings from qualitative studies) to identify, collate, report, align, and select telemedicine options from identified studies and reports, and adopts recommendations for the reporting format of meta-synthesis research. Finfgeld-Connet states that for meta-synthesis, “*Systematic and rigorous methods* [are used] *to identify topically related research reports that provide qualitative findings for analysis. The subsequent analysis of the data goes beyond merely reorganising and recategorising research findings. Newly synthesized concepts are developed*, *and the dynamic relationships among them are fully articulated*” [21]. The intent is to provide an evidence-based foundation from which healthcare decision-makers can “*make context-based decisions and implement situation-specific actions*” [21], in this case in relation to the development of a telemedicine-specific strategy for Botswana.

### 2.2. Data Collection and Sampling

There were five primary sources of data available related to HRI within Botswana:

1. ***Perceptions of healthcare workers and patients***. Although research and pilot projects have been reported in Botswana, for example, by Ndlovu et al. [22], Chavez et al. [23], and Littman-Quinn et al. [24], little was known about perceived telemedicine knowledge, practices, and needs of healthcare workers and patients. A recent survey and key informant-based research provided that insight [13,14]. Surveys of healthcare workers (HCW) and patients in three regions of Botswana had identified their perspectives on priority HRI [14].
2. ***Published telemedicine research from Botswana***. Following the principles of the Preferred Reporting Items for Systematic Reviews and Meta-Analyses (PRISMA) method, studies were identified from searches of four electronic databases (PubMed, Scopus, Web of Science, and Academic Search Complete), searched from 1 January 1990 to 31 December 2022 in April 2023 [25]. The search string was deliberately broad and used specific terms (Botswana, telemedicine, telehealth, ehealth, e-health, mobile health, mhealth, m-health, digital health) linked with the Boolean operators AND or OR. The PubMed search string was (“Botswana” [All Fields] AND (“telemedicine” [All Fields] OR “telehealth” [All Fields] OR “ehealth” [All Fields] OR “e-health” [All Fields] OR “mobile health” [All Fields] OR “mhealth” [All Fields] OR “m-health” [All Fields] OR (“digital” [All Fields] AND “health” [All Fields]))). Inclusion criteria were: addressed eHealth or telemedicine use in Botswana; English language. Titles and abstracts of identified resources were independently reviewed by all three authors for relevance and appropriateness as defined by the inclusion criteria. The full papers of those that met the inclusion criteria were obtained and independently reviewed by all authors to identify the HRI addressed and assess if of national concern (i.e., impacting many Batswana).
3. ***Government reports, policy, and strategy documents***. Desktop searching of Government of Botswana websites and broader searching used Google and Google Scholar was completed during April 2023. Botswanan specific eHealth and telemedicine-related material were identified and downloaded for review (e.g., government health reports; WHO reports).
4. ***Reports from global entities***. Using the authors’ prior knowledge, websites of the WHO and the Institute for Health Metrics and Evaluation were searched for Botswana-relevant documents. Each identified report was downloaded and reviewed.
5. ***Sustainable Development Goals***. These were adopted by all United Nations Member States in 2015 and are acknowledged global targets. They are intended to combat many of the most urgent issues facing mankind, and are recognised targets embedded within many of the goals of the Botswana government.

### 2.3. Data Analysis

Analysis of the documents was complicated by their different foci, with some identifying specific issues, others identifying specific diseases, and others focussing on metrics. Nonetheless, each document identified from the five primary sources of data was reviewed to identify significant HRI (those of national importance, impacting most Batswana), and these HRI data were extracted and charted using Microsoft Excel (v16.0; Microsoft Corporation (Redmond, Washington, DC, USA); Office 365). After collation, each author independently examined the charted data to align, combine, and entitle similar HRI. The process was repeated by the three authors collectively, with discrepancies resolved through consensus.

After charting, it was necessary to prioritise the HRI. The rationale was straightforward. Resource limitations (e.g., human, financial, infrastructural), prevalent in developing countries, mean not every option can or should be pursued. Enforcing choice purposefully limits the options and ensures limited resources are used wisely [8]. Furthermore, the ultimate step (identifying potential telemedicine options to address identified HRI) is simplified when fewer HRI have been identified [13]. However, typically some means of prioritisation is needed and is required during Steps 4 and 7 of the eHSDF [8]. Of the three tools and approaches identified in the eHSDF, the Combined Approach Matrix was considered most appropriate. However, in this study, prioritisation became a major problem, details of which are provided in the Discussion. Ultimately a pragmatic and subjective approach was adopted; those HRI identified by five or more of the primary data sources were designated as priority HRI.

Thereafter authors independently and then collectively identified potential telemedicine solutions for the identified HRI, again with discrepancies resolved through consensus. The personal experience and expertise of the authors, based on over 50 years of collective research and practice in the eHealth field, were used to identify and juxtapose potential telemedicine solutions with each HRI. This allowed the understanding of desirable characteristics of telemedicine solutions to be taken into account, e.g., ease of use, value to users, cost, cultural sensitivity, environmental impact, and technical appropriateness.

### 2.4. Ethics

Approval for this non-invasive desktop study was not required. However, approval for the overall study was granted by the Botswana Ministry of Health and Wellness, Health Research Development Committee (PPME 13/18/1 IX (490)), and the University of KwaZulu-Natal Humanities and Social Sciences Research Ethics Committee (HSS/0872/015D).

## 3. Findings

### 3.1. Sources of Health Related Issues

1. ***Scientifically-based reports from global entities.*** Two reports were retrieved and reviewed [26,27]. These were the country profile for Botswana from the Institute for Health Metrics and Evaluation Global Burden of Disease Study for 2021 [26], and the WHO Country Cooperation Strategy—Botswana [27]. From the former, the top 10 causes of mortality, morbidity, and risk factors were identified. Issues for mortality and morbidity were the same (although the order changed), while some of the risk factors could be aligned with some of the mortality and morbidity issues.
2. ***Perceptions of healthcare workers and patients.*** The list of 14 HRI identified in a previous study [13] was applied to the current study, with the exception of the need for a Laboratory Management Information System which is already addressed in the National eHealth Strategy.
3. ***Government reports, policy, or strategy documents.*** The current versions of seven health-related documents were retrieved and reviewed: Botswana Domesticated SDG Indicators (2018) [28]; National Health Policy 2011 [29]; World Health Organization—Non-communicable Diseases (NCD) Country Profiles, 2018 [30]; Integrated Health Service Plan (IHSP)—A Strategy for Changing the Health Sector for Healthy Botswana 2010–2020 [31]; Vision 2036—Botswana [32]; National Development Plan (NDP) 11—2017–2023 [33]; National Strategic Framework on HIV and AIDS (NSF III) [34]. Each was independently and collectively reviewed for national HRI.
4. ***Botswana telemedicine literature.*** After removal of duplicates and application of the inclusion criteria, 71 resources were identified. Each was independently and collectively reviewed. Of these resources, 24 provided clear evidence to indicate the issue(s) they addressed was of national significance [22,23,24,35,36,37,38,39,40,41,42,43,44,45,46,47,48,49,50,51,52,53,54,55].
5. ***Sustainable development goals (SDGs).*** The original listing of SDGs [56], as well as the previously identified Botswana Domesticated SDG Indicators (2018) document [28] were used. Attention was given to directly health-related SDGs (Goal 3—Good Health and well-being) and to indirectly health-related SDGs including all Goal 6 targets (which related to WaSH programmes; Water, Sanitation, and Hygiene), and 5.2 (which related to interpersonal violence).

Each of the HRI was charted and extracted from the five primary sources of data—expanded to six by separating out morbidity and risk factor data from the Institute for Health Metrics and Evaluation document [26]. All authors independently and collectively aligned, combined, and gave simple descriptors to these HRI (Supplementary Table S1). As a result, a total of 28 HRI were identified. In keeping with the eHSDF principle to focus on the top 20% of identified issues (6 of 28), six ‘priority HRIs’ were separated out, each identified in five of the six sources (Table 1).

### 3.2. Recommended Telemedicine Solutions

The final step was to identify possible telemedicine solutions to address each priority HRI. The potential of telemedicine is diverse and provides a wide spectrum of possible applications. These include telemedicine practice (e.g., provider-to-provider or provider-to-patient consultation for referral or second opinion), in various disciplines (e.g., teleradiology, teledermatology, etc.), for different activities (e.g., clinical service, education, behavioural change), and using various delivery modes (e.g., synchronous, asynchronous, remote monitoring).

In keeping with the eHSDF principle to focus only on the top 20% of identified solutions, those described below have been restricted to only the simplest approaches. For the purpose of this study the categories of interest were ‘activity’ and the ‘delivery mode’. In terms of activity, the priority HRI could be classified into three telemedicine activities: clinical service, education, and behavioural change. For delivery three distinct modes are typically recognised; synchronous (real-time, e.g., videoconferencing), asynchronous (store-and-forward, e.g., e-mail, instant messaging apps), and remote monitoring (e.g., monitoring patients using digital medical devices). In terms of complexity and connectivity requirements, asynchronous is recognised to be the simplest approach. The asynchronous mode is a technique that stores and forwards the patient information (e.g., medical history; images; treatment guidance), educational material (e.g., course notes; examination form; disease information), or behavioural change guidance (e.g., appointment or medication reminders) to another healthcare worker or patient at a later time. Asynchronous telemedicine has been greatly facilitated through the use of mobile phones (mHealth). Potential telemedicine solutions were individually identified, and collectively discussed until consensus was reached for pertinent recommendations (Table 2).

## 4. Discussion

This is the first attempt to employ the eHSDF in a formal way, although not in the national, government-led manner originally envisaged. Nonetheless, application of its principles and process has been successful in allowing meaningful and evidence-based telemedicine solutions to be recommended for Botswana. Preliminary investigations confirmed the need for an evidence-based telemedicine-specific strategy that complements and supports the existing National eHealth Strategy [10]. Such a strategy is necessary to redress the absence of telemedicine initiatives in the National eHealth Strategy, and the current often uncoordinated and unstructured telemedicine interventions that are difficult to scale and sustain—a situation that squanders limited national resources. Pragmatic research studies involving healthcare workers, patients and other eHealth experts clearly revealed the areas of focus that the proposed strategy should address [13,14]. Using this, and evidence from a variety of other sources, six priority HRIs were differentiated (Table 1). Subsequently, specific clinical service, educational, and behavioural change store-and-forward telemedicine options were identified and recommended for incorporation into a telemedicine-specific strategy for Botswana (Table 2).

Prioritisation to identify priority health-related issues was difficult. The GBD study for Botswana effectively addresses disease burden (mortality and morbidity data) and determinants (risk factor data). Therefore, a simple way of prioritising would have been to just consider health issues identified by the GBD study since they are evidence-based and objective. Insight around morbidity identifies what issues the population currently must live with (related to quality of life), and risk factor insight identifies what issues the members of the population could modify in order to improve their quality of life and delay death. As such, giving priority to addressing morbidity and risk factors might be considered most appropriate for any healthcare system. However, this approach would have ignored the other sources of data available—healthcare worker and patient opinion, government documents, available Botswana eHealth literature, and SDGs. Ignoring these might have omitted other important HRIs.

Ultimately, alignment was sought between the GBD morbidity and risk factor categories and the identified health-related issues from other sources, arbitrarily selecting ‘priority’ HRI as those that were identified by at least five of the six data sources. Primary difficulties were related to GBD data focussing on diseases, HCW/patient opinion focussing on health system issues, government documents addressing both disease and health system issues, and the Botswana eHealth literature often focussing on narrow HRI of debatable national significance. The approach taken to identify priority HRI was certainly a compromise, but it did at least allow some of the issues identified by other means to be accommodated and addressed. However, as a result, no issues identified by HCW or patients were included, for example the important issue of shortage of HCW. Furthermore, the approach was not streamlined; for example, in the initial process of data charting, placing the GBD risk factor ‘alcohol use’ in line with ‘interpersonal violence’ (IPV) does ***not*** imply alcohol use is the only HRI of relevance to IPV; similarly, ‘unsafe sex’ is ***not*** the only HRI of relevance to ‘HIV/AIDS’. The process merely aligns those HRI identified from different sources.

Telemedicine has existed for several decades but has never been as widely adopted as expected despite its great potential. For example, telemedicine has been used in a wide range of disciplines to improve access to clinical care and, importantly, specialist care. Similarly, telemedicine solutions have addressed TB, HIV/AIDS, hypertension, and sexual/reproductive health issues. They have also been applied to various aspects of HCW and patient education (awareness, training, in-servicing, continuing professional development/continuing medical education, skills raising), as well as remote data collection/bio-surveillance, and many others. Understanding which solution to adopt, when, why, and how is key.

Reasons for poor uptake vary, but Sony et al. reported a lack of a patient-centric approach or alignment of technology investments with organisational goals and objectives, as well as poor supportive organisational culture, effective leadership, and employee skills [57]. In Middle Eastern countries, barriers were user (doctor and patient) resistance, poor infrastructure, funding, health system quality, and information technology training, as well as cultural, legal, and regulatory barriers [58]. Other barriers have also been noted for developing countries; for example, in Ethiopia infrastructure and costs were the most frequently reported barriers, and staff resistance was noted, while for patients in Pakistan, travelling cost, attitudes, and perceived usefulness were the primary barriers to adoption of telemedicine [59,60].

Choosing a telemedicine solution that overcomes prevailing barriers is difficult. Telemedicine solutions can range from the modest (telephone call, text messaging) to videoconferencing to the very sophisticated (robotic telesurgery). The latter examples can quickly and markedly raise the associated cost, infrastructure needs, connectivity needs, and complexity of the telemedicine setting. This is not ideal, and the literature has for many years supported seeking the simplest, least expensive, and easily scaled approaches [18,19,61]. With regard to Low- and Middle-Income Country (LMIC) settings, the most appropriate mode is asynchronous because it can address the bandwidth constraints as well as the data costs that are comparatively high for developing countries. To a certain extent, asynchronous telemedicine also minimises the impact of the digital divide experienced in many LMIC settings, since many people are familiar with text or e-mail messaging.

It is noted that Botswana has experience with telemedicine, e.g., text messaging for behaviour change, mHealth for store-and-forward telemedicine, and video-conferenced dermatopathology. Therefore, the proposed approaches deliberately leverage Botswana’s cellular network coverage, telemedicine experience, and familiarity of the population with mobile phone use. The recommended technologies are low-bandwidth and are based on asynchronous (store-and-forward) instant messaging, e-mail, or web-based approaches.

As a result, very pragmatic telemedicine approaches have been selected whose specific solution and content can be directed towards one or more of the particular need(s) of each HRI—something the Government of Botswana and other stakeholders would need to clarify. Since more than one solution (technological or otherwise) is possible for any particular HRI, and since telemedicine is dynamic (changing in tandem with technological advancements), any strategy should be periodically reviewed to align with applicable technologies or other options appropriate for future use. Furthermore, as experience is gained by patients, providers, population, and policy-makers, it will become possible and desirable to scale or expand the spectrum of telemedicine delivery modes and activities. For example, as smartphone penetration increases within Botswana, use of the full capability of smart-phone-based applications (such as WhatsApp) may be leveraged, evolving from text messaging, through forwarding of images, to mobile phone-based videoconferencing.

Although simple modes and activities have been proposed, they are effective and will have a broad impact. The practical utility of the study findings manifests in the form of potential telemedicine benefits. If adopted and correctly implemented, Botswana (and perhaps - by emulation - other jurisdictions) will realise potential telemedicine benefits in more systematic, scaled, and sustainable ways. Examples of these benefits include reduced transport costs for both patients and the government (the provider of most health and healthcare services), provision of preventative healthcare for patients, and patient access to healthcare specialist services in underserved areas. Telemedicine also allows for earlier diagnosis and treatment, leading to reduced hospital admissions and costs to the government. It has also been shown that a healthier population provides a healthier and more productive workforce.

The development and implementation of any national strategy is not straightforward. Whilst the government must be involved (ensuring a conducive, safe, regulated, and resourced setting), it must also encourage and support growth and application to flourish from the ‘bottom up’, and not the ‘top down’ in order to lead to valued and sustained use [19,62]. It must also ensure that the correct solution is implemented for the correct purpose. Telemedicine is a component of eHealth whose uses, as noted above, can be focussed or broad, simple or complex, inexpensive or costly. To ensure widespread adoption that leads to successful implementations and encourages future growth, selecting the simplest, easiest to deliver solution has wisdom and merit. This study provides practical guidance whose recommendations can be adopted and pursued to realise a needs-based telemedicine-specific strategy that leads to health system strengthening in Botswana.

### Limitations

This study provides the rationale for pursuing a telemedicine-specific strategy for Botswana, describes the eHSDF exemplar as an approach, and provides recommendations for telemedicine applications. The study has limitations. In the search for resources, only English resources were considered, and other databases may have provided additional resources. More significantly, performance of the eHSDF process in this and prior studies was only emulated as an academic exercise, and not completed as a national, even regional or institutional, activity. If undertaken at such scale, active involvement of a greater number of informed participants and enhanced performance of each step would occur, resulting in a stronger strategy. In addition, the prioritisation process was, as noted, a compromise; a government or other body might approach the process differently and arrive at a different set of priority HRIs, and thereby a different set of suitable telemedicine solutions for consideration. There is no single perfect process for strategy development, therefore the findings of this study, although reasonable, present only one possible set of solutions for a telemedicine-specific strategy for Botswana. Finally, as previously noted, only the Government of Botswana can approve a telemedicine-specific strategy, thus this paper provides only recommendations for consideration. The findings may either be sanctioned or refined at the Government’s discretion.

## 5. Conclusions

The literature indicates that telemedicine solutions to many of Botswana’s health-related issues and needs are possible, and that strategy can guide their implementation. However, without a clear and evidence-based telemedicine-specific strategy to promote and guide the process, telemedicine will be unable to become integral to healthcare delivery in Botswana. As a result, its full potential, particularly as a means of reducing healthcare disparities for those most in need—vulnerable and underserved populations—will not be achieved. In the absence of an adequate focus on telemedicine within the existing National eHealth Strategy, the development of a complementary telemedicine-specific strategy is desirable.

Evidence-based recommendations have been provided to guide the development and implementation of a telemedicine-specific strategy deliberately contextualised to the Botswana setting. Adopting these recommendations would allow Botswana to begin integration of telemedicine into its existing healthcare system and national eHealth strategy. Furthermore, it would result in selection of specific telemedicine approaches and solutions that would provide the most benefit to, and have the most impact on, Batswana.

## Figures and Tables

**Table 1:** The six priority health-related issues (HRI) identified after independent and collective alignment, combining, and entitling. (‘# Sources’ represents how many of the six data sources identified an HRI).

Global Buden of Disease ^a^		HCW/Patient Opinion ^b^	Government of Botswana Documents ^c^	Botswana Telemedicine Literature ^d^	SDG Target ^e^	# Sources
Morbidity	Risk Factors					
HIV/AIDS	Unsafe sex	-	√	HIV/AIDS (delayed CD4 results)	3.3, 3.7	5
Tuberculosis	Tobacco	-	√	TB	3.3, 3.9, 3a	5
Ischemic heart disease (infarction)	High blood pressure; Tobacco; High BMI	-	√	Lack of blood pressure screening	3.4, 3.9, 3a	5
Stroke	High blood pressure; Tobacco; High BMI	-	√	Lack of blood pressure screening	3.4, 3a	5
Diabetes	High fasting plasma glucose; High BMI; Dietary risks	-	√	Diabetic retinopathy detection	3.4	5
Diarrhoeal diseases	WaSH; Malnutrition; Dietary risks	-	√	Monitoring water quality	3.9, 6	5

^a^—sourced from Institute for Health Metrics and Evaluation—Botswana Country Profile (2021) [26]. ^b^—sourced from prior research [13]. ^c^—sourced from multiple Government of Botswana documents (see text)—√ indicates one or more of the Government documents overtly addressed the specific or closely related HRI. ^d^—sourced from multiple documents retrieved from the literature (see text). ^e^—sourced from Botswana Domesticated SDG Indicators (2018) [28]. TB = Tuberculosis. BMI = Body Mass Index. WaSH = Water, Sanitation and Hygiene. CD4 = ‘Cluster of Differentiation 4’ lymphocyte count.

**Table 2:** Examples of telemedicine solutions for each recommended priority health-related issue (HRI).

HRI	Clinical Service	Education	Behaviour Change
HIV/AIDS	IM-based teledermatology, teleradiology, and telepulmonology; e-prescription	IM-based messages (awareness/education)	IM-based messages (medication/appointment reminders; sexual practice)
Tuberculosis	IM-based teleradiology, telepulmonology, and treatment/management; e-prescription	IM/e-mail-based messages (Patient: disease awareness/education) (HCW: in-service skill development/training)	IM-based messages (Patient: TB-DOTS medication reminders/reducing spread)
Ischemic heart disease	IM-based ECG interpretation	IM/e-mail-based messages (disease awareness/education)	IM-based messages (Patient: diet, blood pressure control, exercise)
Stroke	IM/e-mail-based teleconsultation (treatment/management)	IM/e-mail-based messages (Patient: healthy lifestyle, blood pressure control, diabetes control, diet)	IM-based messages (Patient: healthy lifestyle, blood pressure control, diabetes control, diet)
Diabetes	IM/e-mail-based treatment/management; e-prescriptions	IM/e-mail-based educational material (disease awareness/education)	IM-based messages (medication/appointment reminders)
Diarrhoeal diseases	IM/e-mail-based on-call teleconsultation/referral	IM-based educational material (WaSH awareness/education)	IM-based messages (WaSH awareness)

HCW = Healthcare worker; IM-based = Instant messaging-based (e.g., text messaging, WhatsApp); WaSH = Water, Sanitation, Hygiene.

## Data Availability

All data supporting reported results of this desktop study can be found in the publicly available resources cited in the paper.

## Supplementary Materials

The following supporting information can be downloaded at: https://www.mdpi.com/article/10.3390/ijerph20186718/s1, Table S1: Listing of significant health-related issues (HRI) after independent and collective alignment, combining, and entitling. (‘# Sources’ represents how many of the six data sources identified an HRI).

## Lists of Abbreviations

HCW:	Healthcare Workers
HRI:	Health-Related Issues
ICT:	Information and Communications Technologies
eHSDF:	eHealth Strategy Development Framework
