# Supplementary material for: Recommendations for Developing a Telemedicine Strategy for Botswana: A Meta-Synthesis
Source: Int J Environ Res Public Health. 2023 Sep 6;20(18):6718. doi: 10.3390/ijerph20186718 (PMC10530668; doi:10.3390/ijerph20186718)
Supplement: Supplementary file 1 [file ijerph-20-06718-s001.zip › ijerph-2527760-supplementary.pdf]

Table S1: Listing of significant health-related issues (HRI) after independent and collective alignment, combining, and entitling. ('# Sources' represents how many of the six data sources identified an HRI).

GBD [a]		HCW / Patient Opinion [b]	Government of Botswana documents [c]	Botswana telemedicine literature [d]	SDG Target [e]	# Sources
**Morbidity**	**Risk Factors**					
HIV / AIDS	Unsafe sex		√	HIV / AIDS (delayed CD4 results; dermatological care; antenatal ART)	3.3, 3.7	5
Tuberculosis	Tobacco		√	TB screening / referral	3.3, 3.9, 3a	5
Ischemic heart disease (infarction)	High blood pressure; Tobacco; High body-mass index		√	Lack of blood pressure screening	3.4, 3.9, 3a	5
Stroke	High blood pressure; Tobacco; High body-mass index		√	Lack of high blood pressure screening	3.4, 3a	5
Diabetes	High fasting plasma glucose; High body-mass index; Dietary risks		√	Diabetic retinopathy detection	3.4	5
Diarrheal diseases	WaSH; Malnutrition; Dietary risks		√	Monitoring water quality	3.9, 6,	5
Neonatal disorders			√	High child mortality	3.2	4
Lower respiratory infection	Tobacco; Malnutrition; Air pollution		√		3.4, 3.9, 3a	4
		Shortage of HCWs;	√	Shortage of HCWs; Poor access to specialised healthcare	3c	4
Road injuries	Alcohol use		√		3.6	4
Interpersonal violence	Alcohol use		√		3.5, 5.2	4
		Prevalence of diseases (TB, blood pressure, cancer, HIV/ AIDS, malaria, CVD)	√	Prevalence of TB, HIV, AIDS; Tracking infectious diseases.	3.3, 3.4	4

		Inefficient / poor referral system	√	HCW information exchange; Poor access to specialised healthcare		3
		Lack of diagnostic and case management skills	√	Poor access to health education.		3
		Costs of accessing healthcare facilities and services	√	…	3.8	3
		Lack of medical equipment		Scarcity of resources	3.8	3
		Loss of patients to follow-up or monitoring	√			2
		Lack of medical and drug supplies		…	3.8, 3b	2
		Delayed reporting of laboratory results		Delayed reporting of CD4 results; LIMS management challenges		2
		Lack of ICT knowledge		Access / exchange healthcare information		2
		Congestion / over-crowding at healthcare facilities				1
		Transport shortages				1
		Increased patient workloads				1
				Rehabilitation issues		1
				Clinical information needs		1
				Cervical cancer screening.		1
				Specialist resources		1

				(Dermatology, Radiology)		
				eHealth interoperability issues		1

[a] - sourced from Institute for Health Metrics and Evaluation – Botswana Country Profile (2021) [26]
[b] - sourced from prior research [13]
[c] - sourced from multiple Government of Botswana documents (see text) - √ indicates one or more of the Government documents overtly addressed the specific or closely related HRI
[d] - sourced from multiple documents retrieved from the literature (see text)
[e] - sourced from Botswana domesticated SDG indicators (2018) [28]TB = Tuberculosis
BMI = Body Mass Index
WaSH = Water, Sanitation and Hygiene
CD4 = *'Cluster of Differentiation 4' lymphocyte count*
LIMS = Laboratory Information Management System
HCW = Healthcare Worker
NCD = Non-Communicable Disease
CD = Communicable Disease
